# Unusual Circulation Patterns of the Rias Baixas Induced by Minho Freshwater Intrusion (NW of the Iberian Peninsula)

**DOI:** 10.1371/journal.pone.0112587

**Published:** 2014-11-17

**Authors:** Magda Catarina Sousa, Renato Mendes, Ines Alvarez, Nuno Vaz, Moncho Gomez-Gesteira, João Miguel Dias

**Affiliations:** 1 CESAM, Departamento de Física, Universidade de Aveiro, Campus de Santiago, Aveiro, Portugal; 2 EPhysLab (Environmental Physics Laboratory), Universidade de Vigo, Facultade de Ciencias, Ourense, Spain; Centro de Investigacion Cientifica y Educacion Superior de Ensenada, Mexico

## Abstract

The Minho River, situated 30 km south of the Rias Baixas, is the most important freshwater source flowing into the Western Galician coast (NW of the Iberian Peninsula). The buoyancy generated by the Minho estuarine plume can reverse the normal circulation pattern inside the Rias Baixas affecting the exchange between the Rias and the ocean, changing the input of nutrients. Nevertheless, this inversion of the circulation patterns is not a well-monitored phenomenon. The only published results based on *in situ* data related to the presence of the Minho River plume inside the Rias de Vigo and Pontevedra correspond to an event measured on spring 1998. In this case unexpectedly higher inflow surface current velocities were found at the Ria de Pontevedra, located further away from Minho River. Thus, the main aim of this study is to research the main factors inducing this unusual pattern on the circulation of the Rias de Vigo and Pontevedra. A numerical model implementation of MOHID previously developed, calibrated, and validated for this coastal area was used. Several scenarios were performed in order to explain the individual effect of the Minho River, rivers discharging into each Rias, and estuarine morphology changes. According to the model results, the Minho River discharge is a key factor in the establishment of the negative circulation, while small rivers inside the Rias slightly attenuate this circulation. The negative circulation was stronger in Ria de Pontevedra independently of the distance of this coastal system from the Minho River mouth, showing that morphologic estuarine features are the main factor justifying the different local circulation patterns.

## Introduction

Major rivers inject freshwater onto the adjacent shelf where mixing of these river plumes takes place, affecting the transport and transformation of dissolved and particulate materials in the coast. The coastal waters freshening due to river runoff is one of the mechanisms that control the density inside the estuaries located up north or south of the river mouth, modifying the circulation and the macronutrients concentrations available for production [1]–[3]. Numerous studies conducted over diverse coastal systems around the world have revealed a major interest in the ecological consequences of freshwater intrusion on nearby estuaries (e. g. Columbia River [1], Delaware River [3] or Mississippi River [2]). These studies showed that the river plumes from major rivers produce currents in the surface layers of neighbor estuaries, affecting local species and marsh plants.

**Figure 1:**
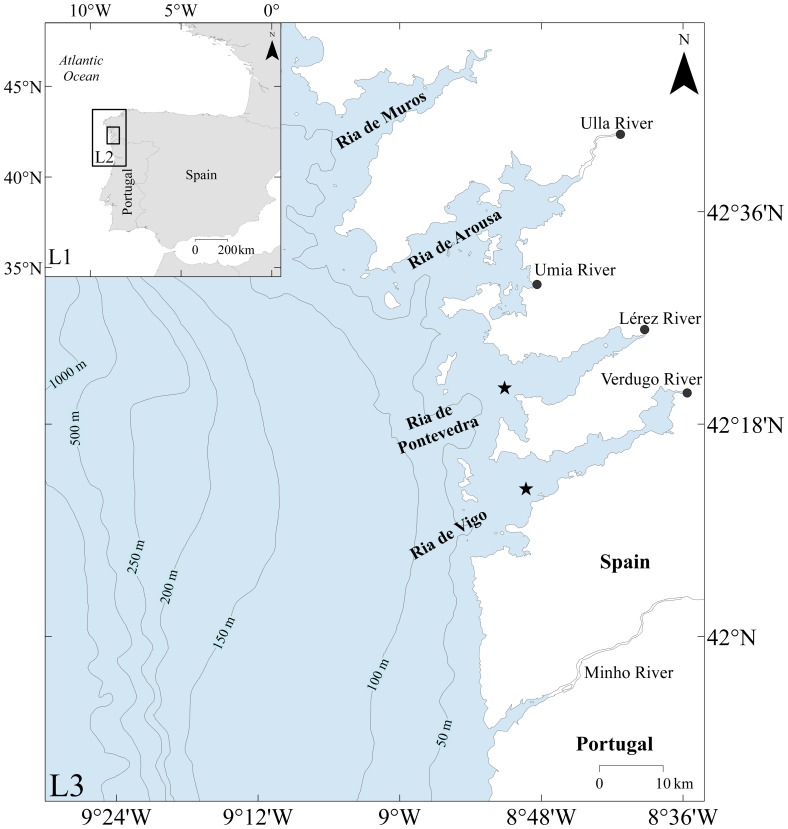
Nested grids and Rias Baixas map.

The NW coast of the Iberian Peninsula is characterized by the presence of several embayments, usually called “Rias”. The term “ria” defines a coastal shape correspondent to re-entrance, resultant from the submersion by the sea of the terminal zone of a fluvial network [4]. The Rias Baixas (Figure 1) are located in the northern limit of the Eastern North Atlantic Upwelling System [5]. They are heavy populated ecosystems of enormous potential, both economical (e.g. exploration of marine resources: fisheries, aquaculture and fishing industry) and social (e.g. tourism: beaches and natural beauty). Wind driven coastal upwelling is the main recognized local source of primary production, supporting the high fishery and aquaculture yields [6], [7].

Located 30 km southward, the Minho River is the most important river flowing near this coastal region. It has a catchment area of 17080 km^2^ and an annual average discharge of 300 m^3^s^−1^. The monthly average discharge oscillates between 100 m^3^s^−1^ in August and 800 m^3^s^−1^ in February [8]. Essentially, during high river discharge and southerly wind conditions, its buoyant plume generates an important salinity decrease at the Rias Baixas mouth, reversing the normal circulation pattern [9]–[11]. This reverse in the circulation pattern (negative circulation) is characterized by the intrusion of less dense coastal waters at the surface, which accumulate within the Rias and finally flow towards the ocean through the bottom [9], [10], [12]–[14]. This inversion of the normal circulation patterns is not a well-documented phenomenon. The infrequent sampling in the inner and outer part of the Rias complicates the characterization of such events through analysis of field measurements. In fact, the only published results related to the presence of the Minho River plume inside the Rias Baixas correspond to an event measured on spring 1998 when a high Minho River discharge was reported as well as southerly wind conditions crucial to spread the river plume towards the Rias Baixas, generating a negative/reverse circulation inside them.

Alternatively, the use of numerical models constitutes a reliable and useful tool to analyze this type of events. The numerical models are used worldwide in order to quantify and understand different processes occurring in coastal waters [15]–[19]. They allow to investigate hypothetical scenarios (e.g. changes in wind and river discharge), which observations alone cannot provide. In addition, they afford the capability to study processes independently. Using a numerical model, Sousa et al. [14] reproduced the experimental campaign conducted on spring 1998 inside the Rias Baixas. They observed that under northward winds, a continuous moderate Minho River discharge (>700 m^3^ s^−1^) is enough to produce the negative circulation pattern in Rias de Vigo and Pontevedra, reducing the importance of the existence of specific events of high river runoff. However, unexpectedly, higher inflow surface current velocities were found at the Ria located further away from Minho River (at Ria de Pontevedra rather than at Ria de Vigo, which is close to Minho River)). However, the explanation of these velocity differences was not evaluated in this previous study.

Considering these issues the present study aims to research the main reasons that induce this unusual pattern in the circulation in Rias de Vigo and Pontevedra on May 1998 event, through the application of a numerical model using a downscaling approach [11], [14]. Several hypotheses were formulated to justify this pattern, namely the impact of different freshwater inflows at the head of each Ria, comparing to the impact of the Minho River and the Rias morphology, that have different width and depth and mouths with different features that may constrain the water exchange with the shelf. To test these hypotheses and explain the different current velocities observed at both Rias were defined several numerical scenarios. In the first, the real conditions corresponding to May 1998 are considered. The second and third scenarios are used in order to evaluate the influence of the Minho River and the rivers flowing into the Rias. The influence of estuarine morphology is evaluated in the last scenario, interchanging the location of the Rias de Vigo and Pontevedra.

The paper is organized as follows. In the second section, the description of study area is presented. The third part presents the general overview of the numerical model as well as the numerical experimental design. Results and discussion can be found in the fourth part. Finally, conclusions are drawn in the fifth part.

### Study area

The study region comprises only two Galician Rias: the Rias de Vigo and Pontevedra, which are the closest estuarine regions located up north of the Minho Estuary.

The Ria de Vigo, which is the most meridional of the Rias Baixas is located around 30 km northwards of the Minho River mouth (Figure 1). This Ria is 32.5 km long, presenting an NE-SW direction, 1 km wide in its inner part (NE) and 10 km at the mouth of the Ria (SW). The mean width and depth are 4.8 km and 21 m, respectively. The mouth of this Ria is divided in two entrances defined by a set of islands, which allow the connection to the open sea. The northern entrance is 2.8 km wide and has a maximum depth of 25 m and the southern one is 5.1 km wide and 45 m depth. The Verdugo River is the main tributary of freshwater into Ria de Vigo with a catchment area of 333 km^2^. Its annual mean annual discharge is 13 m^3^s^−1^ with a greatest seasonal variability (values range from 120 m^3^s^−1^ in winter to 1 m^3^s^−1^ in summer) [20].

The Ria de Pontevedra is located northward of Ria de Vigo (Figure 1). This Ria is oriented in the NE-SW direction. The mean depth and width are 31 m and 3.8 km, respectively. It is also connected to the ocean by two entrances. The northern one is narrower (3.6 km) and shallower (15 m), while the southern mouth is wider (7.3 km) and deeper (60 m). The Lérez River is the main freshwater source that flows into Ria de Pontevedra. This river has a catchment area of 450 km^2^ and an annual mean discharge of 27.5 m^3^s^−1^. The monthly mean discharge oscillates between 2 and 80 m^3^s^−1^ and follows a similar pattern to the rainfall, as dams do not control its runoff.

Concerning the tidal forcing, both Rias are estuarine-like systems that have a semi-diurnal tidal regime. The tidal range varies between 2 and 4 m (mesotidal regime) and a significantly low Form Number of 0.25 is found [21]. The Rias behave normally as partially mixed estuaries, with positive residual circulation and two layer circulation pattern, with surface outflow and inflow on the bottom [22]–[24], although a reverse circulation may be found as previously referred.

## Methodology

### Numerical model

In this work was used the model MOHID (www.mohid.com), previously implemented and validated for the Rias Baixas by Sousa et al. [14].

The model implementation consists in a set of three-one-way nested models (Figure 1). The first domain is a barotropic tidal driven model using the FES2004 (Finite Element Solution) global solution as forcing [25]. It includes the whole Iberian Peninsula coast (L1, Figure 1), with a horizontal resolution of 0.06°. The ocean boundary conditions were given in cascade starting at the first level. Therefore, the 2D barotropic model is only used to predict the external tidal conditions necessary to feed the L2 (Figure 1) baroclinic model with surface elevation.

The second and third domains are baroclinic models (L2 and L3, Figure 1), being the second domain a coarse representation (horizontal resolution of 0.02°) of the Rias Baixas and adjacent coastal area, while the third includes the same area with a higher resolution (horizontal resolution of 0.005°) and it is directly coupled to L2 at the open boundaries. A *z*-level vertical discretization was adopted, with L2 and L3 having 46 and 42 vertical layers, where the bottom 39 (L2) and 35 (L3) were in Cartesian coordinates and the top 10 m were 7 sigma coordinate layers.

To obtain the initial ocean stratification, L2 and L3 were forced at the open boundaries with monthly mean climatologies profiles from Levitus [26], [27].

The surface boundary condition was imposed using hourly high-resolution results from the Weather Research and Forecasting model with a spatial resolution of 4 km. At the surface, heat fluxes were imposed with parameterizations similar to those described by Chapra [28]. The sensible and latent heat fluxes were calculated using the Bowen and Dalton laws, respectively [28]. For the bottom boundary condition, shear friction was imposed, assuming a velocity logarithmic profile.

The freshwater inputs inside Rias Baixas and the Minho estuary outflow were considered as landward boundary conditions (L3). This last was imposed offline in L3 as momentum, water and mass discharges to the coastal model. This outflow was computed with a 0.0065°–0.001° horizontal resolution estuarine model for the inner part of the Minho River [14].

The model was run from November 1997 to May 1998 (the first six-months were considered as spin-up).

A more detailed description of this numerical implementation can be found in Sousa et al. [14].

### Model scenarios

**Figure 2:**
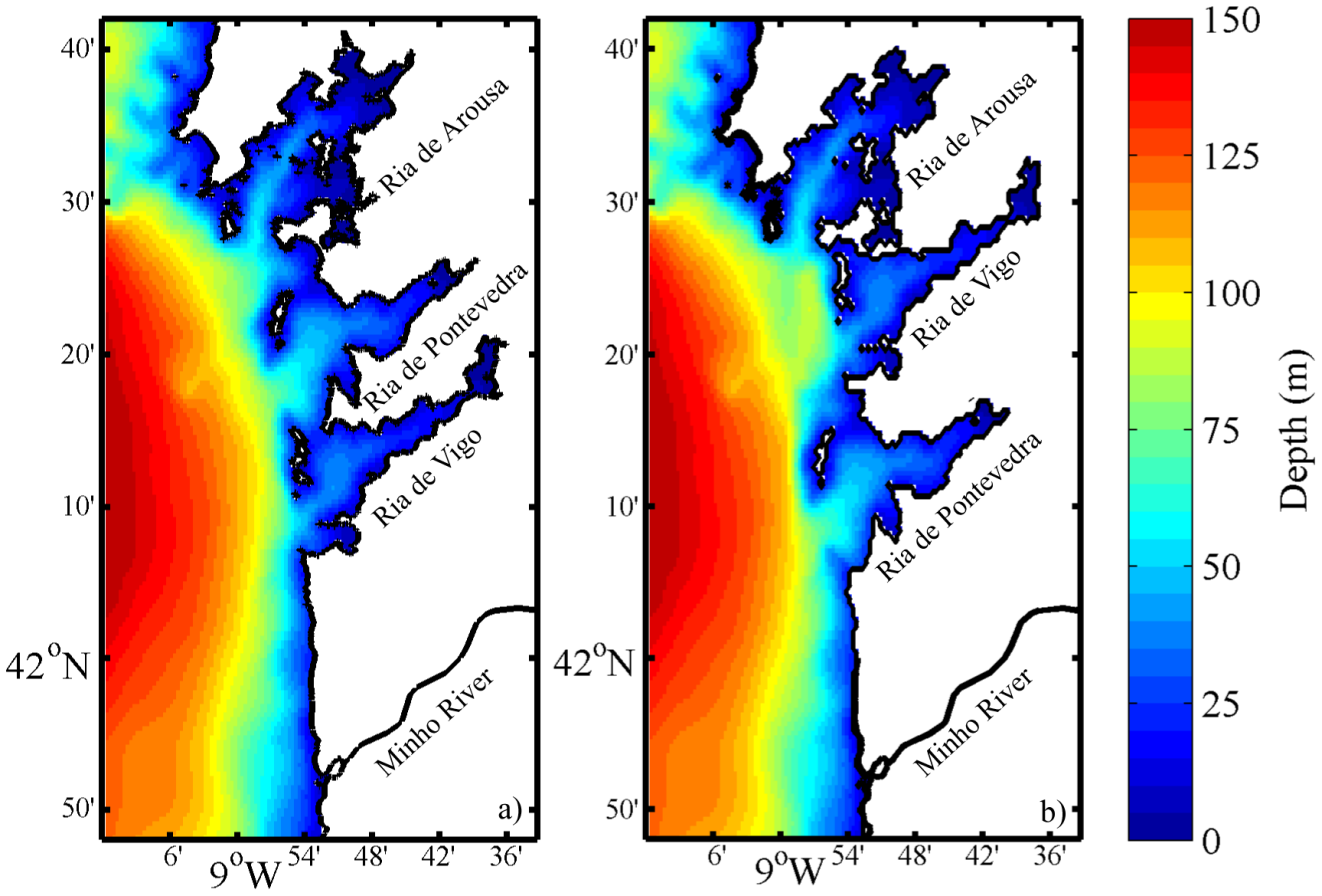
Reference (a) and interchanged (b) bathymetric grids. To simplify visual comparison the Verdugo and Lérez rivers were multiplied by 5.

Four simulations were carried out in order to evaluate the main factors that induce the unusual pattern on May 1998 (Table 1). In the first scenario, the real conditions corresponding to May 1998 were considered (called reference scenario hereafter). The second simulation was run without Minho River discharge during the whole simulation period (called without Minho River scenarios hereafter), but including the freshwater discharges inside each Ria. In the third scenario, the influence of the rivers flowing into the Rias was analyzed. Thus, no discharges from rivers inside the Rias were considered (called without inside rivers scenario hereafter), but was considered the Minho River discharge. With respect to the influence of estuarine morphology, a new bathymetric grid was built interchanging the position of the entire estuarine areas in study, from the mouth to the easternmost grid point of both Rias (inclusive the islands) (Figure 2). This new grid was considered in the last scenario (called interchanged Rias scenario hereafter), where the Ria de Pontevedra is nearest to Minho River instead of the Ria de Vigo (Figure 2b). Despite this interchanging, each Ria preserves its freshwater input, i.e. Verdugo River discharges to the Ria de Vigo and Lérez River to the Ria de Pontevedra.

**Table 1:** Model runs overview.

*Name*	*Minho River discharge*	*Verdugo River discharge*	*Lérez River discharge*
Reference	X	X	X
Without Minho river	-	X	X
Without Inside river	X	-	-
Interchanged Rias	X	X	X

**Figure 3:**
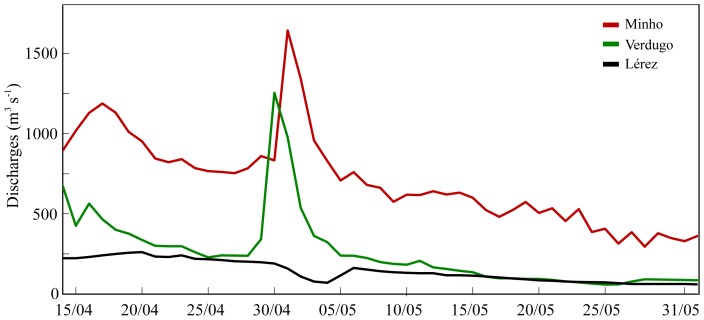
Time series of Minho, Verdugo and Lérez River discharges from 15 April to 31 May 1998.

Three rivers were imposed as land boundaries for the simulations period: Minho, Verdugo, and Lérez (Figure 1). The Minho River discharge shows an atypical pattern with high values during early May (Figure 3). The maximum value is about 1600 m^3^s^−1^. After this date, a continuous decrease of the river discharge is observed. The Verdugo and Lérez Rivers present a similar pattern with a small runoff decrease in late May, except between 29 April and 3 May when the Verdugo River shows high values (250 m^3^s^−1^, Figure 3).

To accomplish the main objective of this work, horizontal maps of the surface density were taken in the instant that Minho River plume reaches the Rias de Vigo and Pontevedra. They were analyzed for the reference and interchanged Rias scenarios. Moreover, the temporal evolution of vertical estuarine circulation at stations located at mouths of Rias de Vigo (∼30 m deep) and Pontevedra (∼35 m deep) (Figure 1, stars) were calculated for the same scenarios. The residual current was also computed for all scenarios by time averaging the hourly transient values from current velocities at mouth of both Rias (Figure 1, stars) over a period of 13 hours, corresponding to the plume intrusion.

## Results and Discussion

In this section, the impact of the Minho River plume and the Rias de Vigo and Ponetevedra main freshwater sources in the establishment of negative circulation in the Rias will be evaluated. In addition, the impact of changes in the position of the Rias (e.g. estuarine morphology *versus* distance to Minho River) on estuarine circulation will be evaluated for the period of May 1998.

### Real conditions in May 1998

**Figure 4:**
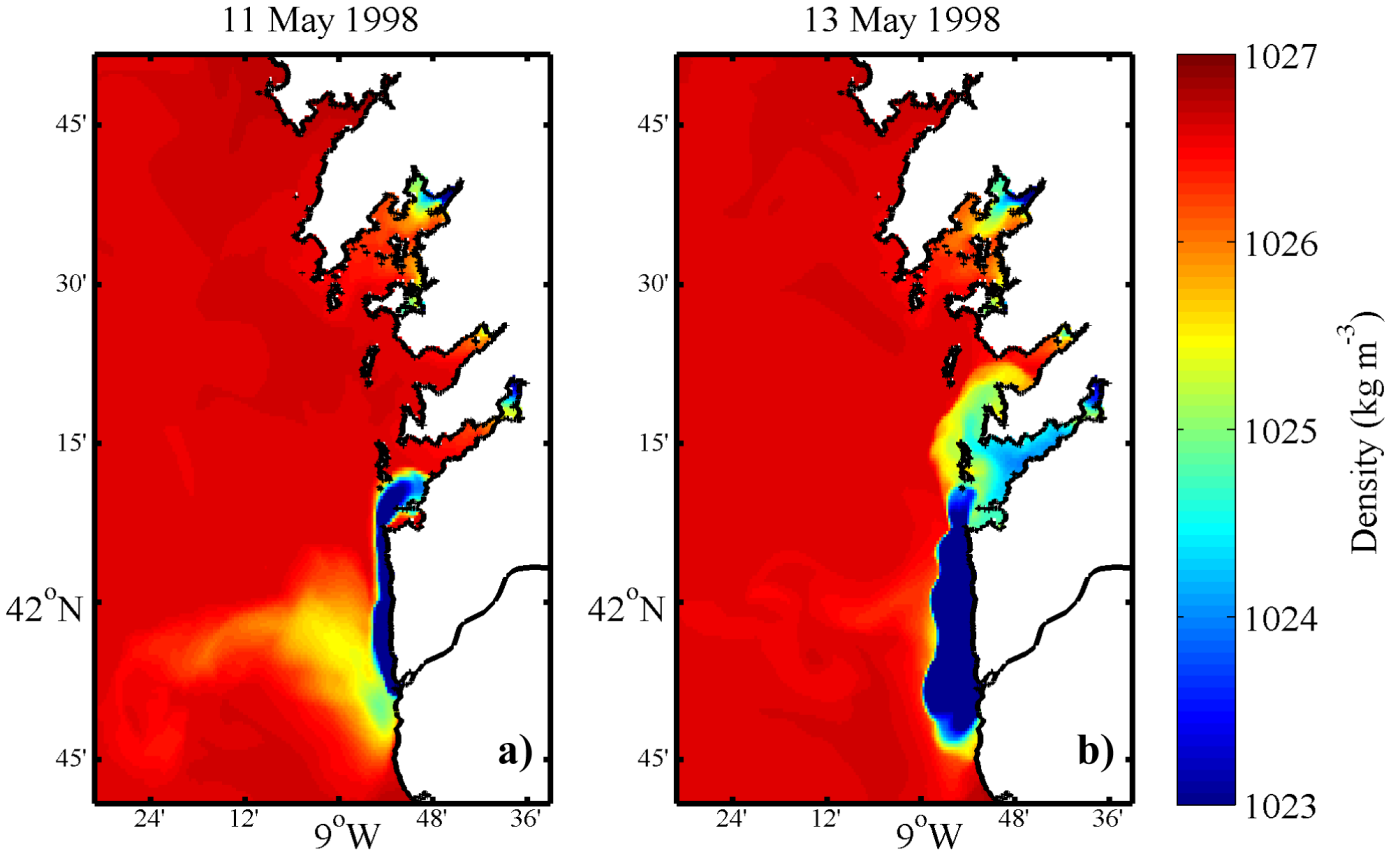
Surface density maps, considering the reference scenario.

Surface density maps show a northward spread of the Minho River plume reaching the Ria de Vigo on 11 May (Figure 4a). During the following days, the northward displacement of the plume continued and the plume intrusion is observed in Ria de Pontevedra on 13 May (Figure 4b). This situation generates an unusual surface density pattern influencing the physical properties within the Rias, reversing the normal density pattern characterized by the presence of saltier water at the outer part of the estuary and freshwater at the middle-inner part. These unusual inflows of freshwater are critical from an ecological point of view, affecting the exchange between the Rias and the ocean, and therefore changing the normal input of nutrients [9], [10]. Thus, this intrusion into the Rias can impose a control on new production within the estuary environment and changes in the mortality of halophyte organisms inside an estuary [29]. Changes in estuarine density gradients can also affect higher trophic levels, particularly the distribution of fishes in estuaries [2]. These consequences demonstrate the high interest in characterizing these types of events in detail.

**Figure 5:**
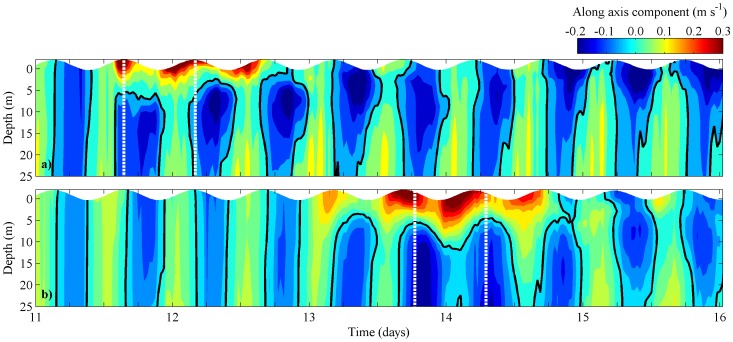
Temporal evolution of vertical profiles of along velocity component at stations located at mouths of Rias de Vigo (b) and Pontevedra (d), considering the reference scenario. Black line corresponds to 0 m s^−1^.

In terms of estuarine circulation, the existence of an important external freshwater supply at the mouth of an estuary can generate the reversal of the normal estuarine circulation [14]. Thereby, the temporal evolution of vertical estuarine circulation at stations located at mouths of Rias de Vigo and Pontevedra (Figure 1, stars) between 11 and 16 May 1998 is also studied through the analysis of the along axis velocity component vertical profiles (Figure 5). In Ria de Vigo (Figure 5a), between 11 and 13 May, a negative circulation (upstream circulation) at upper layers (0–8 m) is observed. There is a landward water movement with highest velocities (0.25 m s^−1^) between 0–2 m depth and a seaward movement at bottom layers. Positive surface velocities are more intense than the negative bottom ones, corresponding to the intrusion of the Minho estuarine outflow into the Ria de Vigo [14].

A similar pattern is recognized in Ria de Pontevedra (Figure 5b), although the surface water moving from ocean to land is observed later (13–15 May). In addition, this unusual circulation is more intense (0.30 m s^−1^) than in Ria de Vigo, with positive values until 10 m depth. In fact, around 13–14 May surface water moving landward was observed during a tidal cycle. Freshwater supplied by the Minho River generates this unusual circulation, which tends to stop water exchange between the Rias and the shelf, increasing its residence time and consequently changing the water quality [10]. This inversion on estuarine circulation may introduce into the Rias dinoflagellate blooms, which are usually generated in coastal waters, decreasing the abundance of marine species in this area [30].

Due to the ecological importance of estuaries and other coastal ecosystems, external freshwater intrusion inside estuaries has also been studied worldwide, as usually as consequence of remote river plumes spreading. As an example, Monteiro and Largier [31] and Hickey et al. [32] showed that external sources of freshwater can have an higher impact on the mean baroclinic circulation inside the Saldanha and Willapa bays than usual estuarine processes (ex: changes in river flow or neap-spring variation in mixing). These results are consistent with the results presented here for the Rias de Vigo and Pontevedra.

### Effect of Minho River and rivers flowing into the Rias

**Figure 6:**
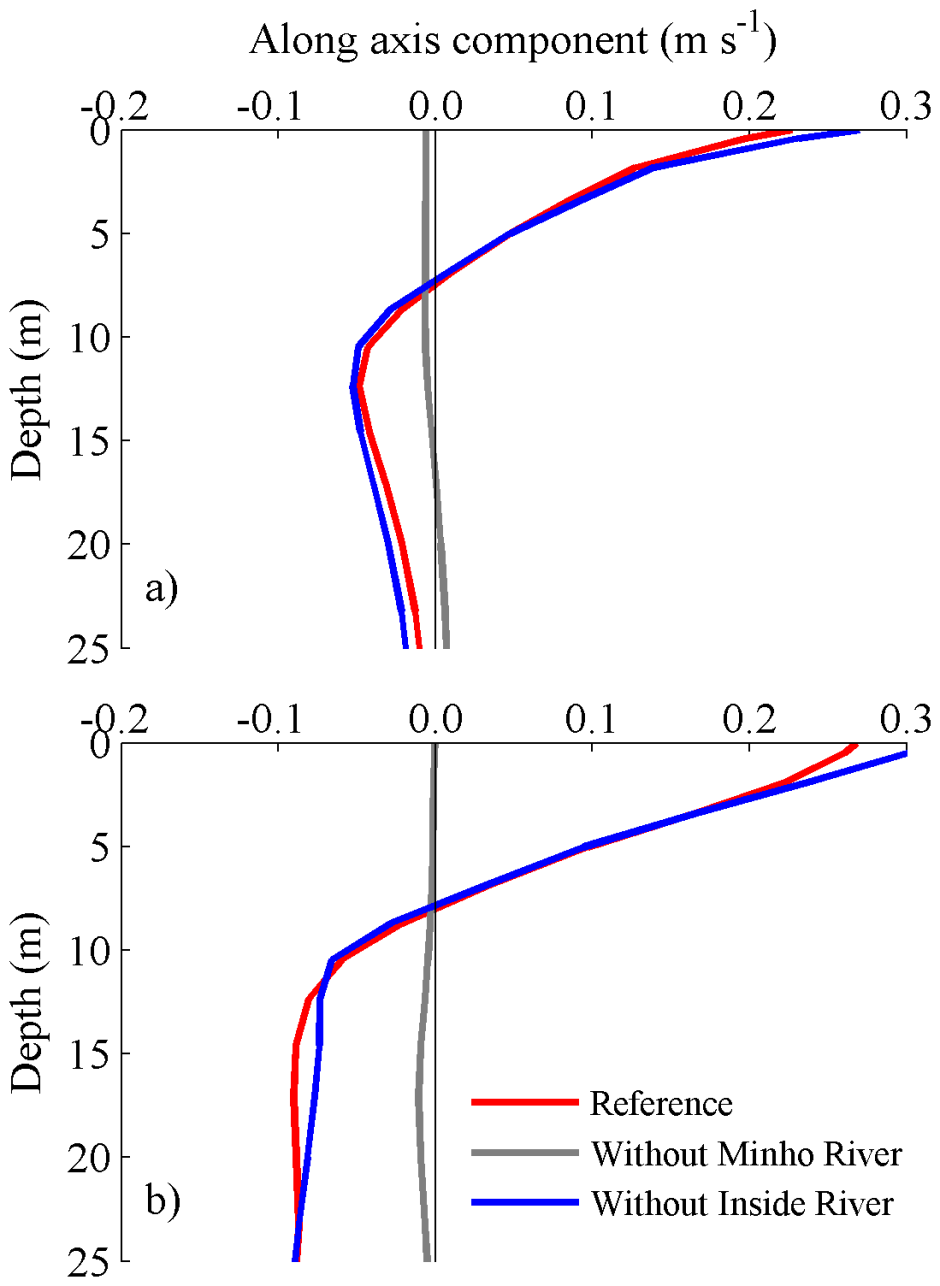
Residual current profiles during the plume intrusion (dashed lines, Figure 4) at stations located at mouths of Rias de Vigo (a) and Pontevedra (b), considering the different scenarios.

Residual circulation is crucial in order to determine the long-term transport in estuaries and therefore a key parameter in the dynamical behavior during the intrusion of the Minho estuarine plume on Rias Baixas. Thus, the residual currents induced by the plume intrusion are presented in Figure 6 for the reference, without Minho River, and inside rivers scenarios. The pattern of the vertical structure of velocity is very similar in both Rias concerning the reference scenario (Figure 6, red line). The inversion of circulation occurs at the same depth (∼10 m). Both in the surface and in the bottom, the highest velocities are observed at the Ria located further away from Minho River (Ria de Pontevedra). Without Minho River discharge (Figure 6, grey line), lower residual velocities are observed for both Rias, showing that the Minho River discharge is the main responsible for the inversion of the circulation pattern and for the non-tidal estuarine transport of dissolved and particulate materials inside the Rias. For the third scenario (removing the rivers flowing into the Rias, Figure 6, blue line), the residual current profile is similar to the one corresponding to the reference simulation. A greater difference is observed on the surface layer for both Rias. The velocity is higher (about 0.02 m s^−1^) than for the reference scenario, showing that if rivers flowing into the Rias are neglected, the Minho estuarine plume would affect more significantly the surface circulation of the Rias. These results also demonstrate that the unusual higher surface current velocities found at the Ria de Pontevedra are not induced by the internal freshwater discharges characteristics.

These results show that the Minho River discharge plays an important role in the establishment of the negative circulation inside the Rias. On the contrary, the small rivers that flow through the interior of the Rias do not influence significantly the inversion of the circulation. These findings are in accordance with the results reported for other regions worldwide, as Willapa Bay [1], where under favorable downwelling winds, the plume from Columbia River forces an axial gradient reversal inside the bay.

### Effect of estuarine morphology

Comparing the results for each Ria independently, it is observed that the integrated residual currents, both at surface and bottom, are greater in Ria de Pontevedra (that is further way of Minho River) than in Ria de Vigo for all scenarios. For example, in the reference scenario (Figure 6, red line), the surface velocity in Ria de Pontevedra is 0.18 m s^−1^ (Figure 6b) while in Ria de Vigo is 0.12 m s^−1^ (Figure 6a). This could (or not) be related to differences in the morphology of each Ria (width and depth as well as different mouth geometries). In order to better analyze this situation the influence of the estuary morphology was evaluated by interchanging the location of Rias de Vigo and Pontevedra (Figure 2b). After that, the methodology adopted for the results presentation in this scenario is analogous to those shown previously.

**Figure 7:**
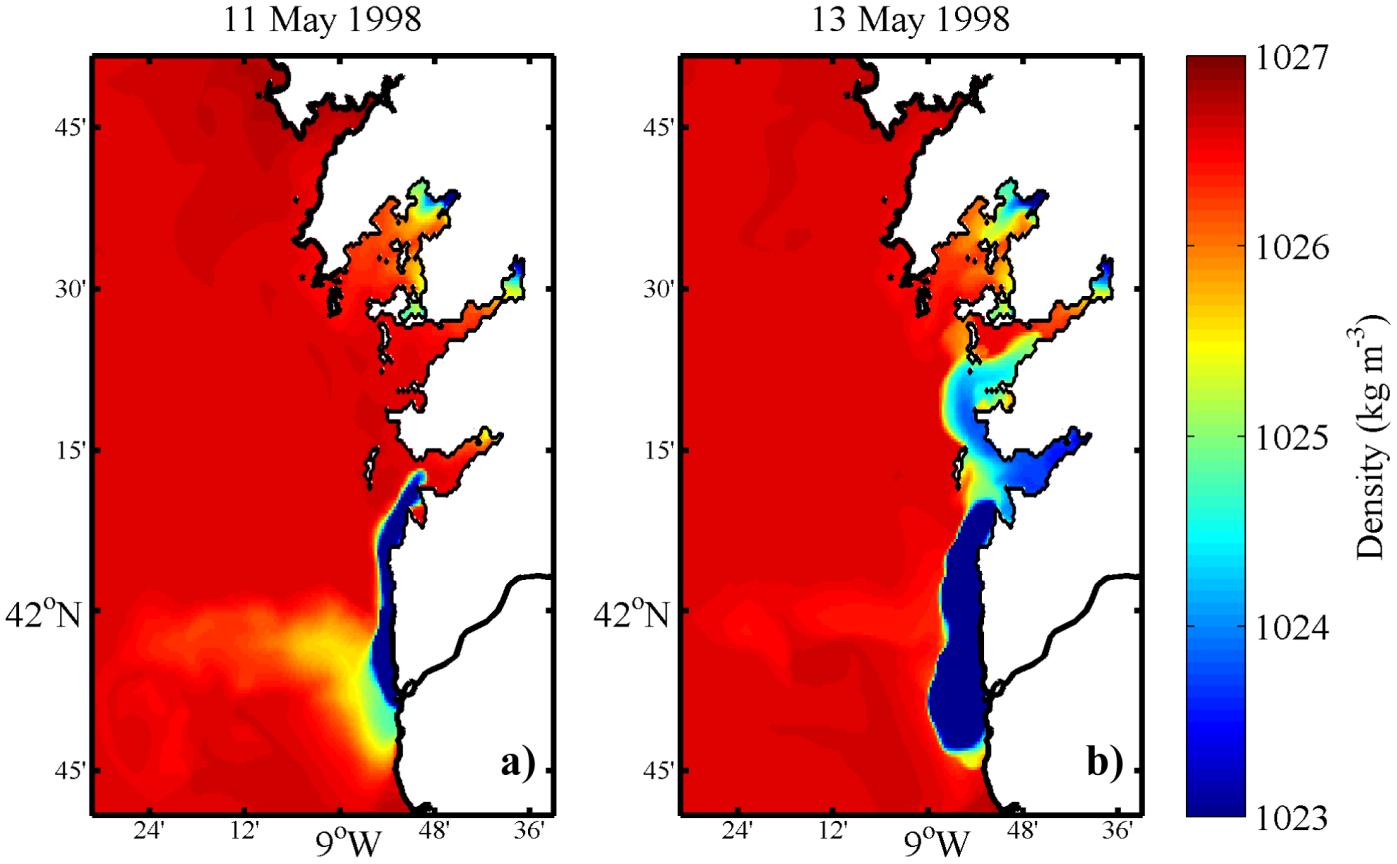
Surface density maps, considering the interchanged Rias scenario.

Generally, the surface density maps are very similar to the reference scenario, with the plume intrusion reaching the Rias at the same period (Figures 7a and 7b).

**Figure 8:**
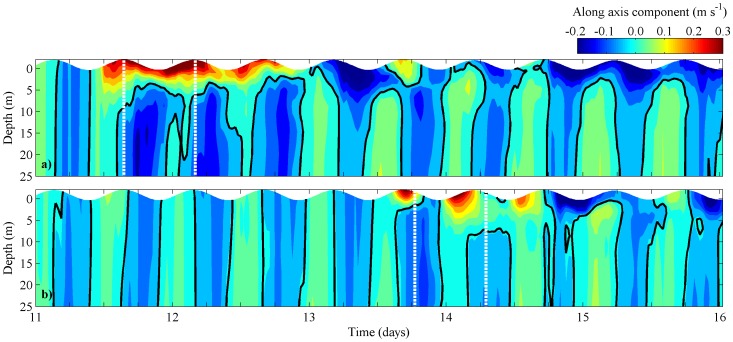
Temporal evolution of vertical profiles of along velocity component at stations located at mouths of Rias de Pontevedra (a) and Vigo (a), considering the interchanged Rias scenario. Black line corresponds to 0 m s^−1^.

**Figure 9:**
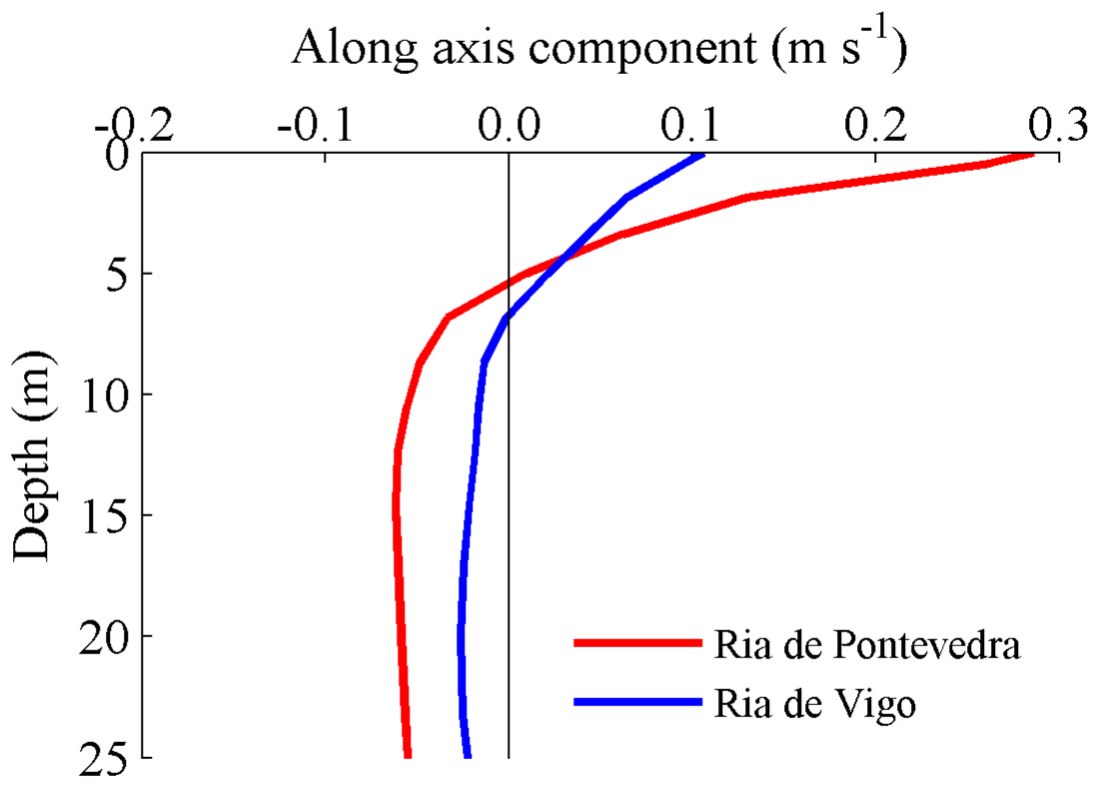
Residual current profiles during the plume intrusion (dashed lines, Figure 7) at stations located at mouths of Rias de Pontevedra and Vigo, considering the interchanged Rias scenario.

For this scenario, the temporal evolution of the vertical profiles of along axis velocity component at the mouth of Rias de Pontevedra and Vigo is also calculated (Figures 8a and 8b). The pattern found for Ria de Pontevedra (Figure 8a) is very similar to that found for the real scenario (Figure 5b), with positive velocities at surface layers (0.25 m s^−1^) and negative values near bottom (–0.10 m s^−1^), corresponding to the intrusion of the Minho estuarine outflow. In Ria de Vigo (Figure 8d), the surface velocity is weaker (0.15 m s^−1^) than for the reference scenario (Figure 5a), and in this case the surface water moving landwards only lasts one tidal cycle. In this scenario, the negative circulation is also stronger (in terms of velocity intensity) at Ria de Pontevedra (Figure 8a), indicating that the morphologic estuarine changes are particularly linked to the intensity of the circulation pattern. This is corroborated by the values of the residual currents generated during the plume intrusion in this scenario (Figure 9). Surface velocities are twice higher for Ria de Pontevedra (0.09 m s^−1^) than for Ria de Vigo (0.04 m s^−1^), showing that the estuarine morphology influences the intensity of the negative circulation.

As expected, these results reveal that the greater density gradients are visible in the Ria located close to the Minho River mouth for all studied scenarios (including the interchanged one). However, in terms of residual circulation, the morphology of each Ria become a critical factor, since residual flows are highly influenced by depth, width and mouth geometry of an estuary [33], [34]. Here, the Ria de Pontevedra always presents higher velocities than the Ria de Vigo, showing the importance of the estuarine morphology. The Ria de Pontevedra is slightly narrower and deeper than Ria de Vigo. As the water content averages for both Rias is similar (3.12 km^3^ for Ria de Vigo and 3.45 km^3^ for Ria de Pontevedra, Alvarez et al. [35]), the morphology entrances is the crucial factor the plume propagation inside the Rias. Regarding only the Ria de Pontevedra, the residual surface velocity in the interchanged scenario is slightly higher (0.28 m s^−1^) than the reference one (0.26 m s^−1^). This difference can be attributed to the higher buoyancy forcing (>density gradients) which squeezes the plume to the surface, increasing the horizontal current velocity towards the interior of the Ria. Warrick and Stevens [36] also showed in their work in Elwha River in the Washington coast that the topographic features influence the buoyant plume behavior, affecting the coastal circulation and sediment transport.

## Conclusions

The main aim of this work was to research the main reasons that induce unusual higher inflow surface current velocities at the Ria located further away from Minho River when the normal estuarine circulation is reversed, through a numerical model MOHID. The individual effect of the Minho River discharge, rivers tributaries flowing into the Rias and estuarine morphology in the Rias Baixas circulation was analyzed simulating several scenarios.

The results obtained from this study have shown that the freshwater intrusion of the Minho River into Rias de Vigo and Pontevedra was the main responsible for the negative circulation pattern and unusual density gradient in May 1998.

Without rivers flowing into the Rias, the Minho estuarine plume affects more significantly the Rias surface circulation, showing that small rivers inside Rias slightly attenuate the negative circulation.

Independently of the location of both Rias, the negative circulation was always stronger in Ria de Pontevedra, showing that the proximity with the Minho River is not the main factor justifying this pattern. Instead, the morphologic estuarine characteristics determine the unusual circulation pattern found.

In summary, the model predictions reproduce accurately the circulation pattern on May 1998 event. The obtained model results indicated that the Minho River plays an important role in the density patterns in Rias de Vigo and Pontevedra and that their morphology is the main factor for the establishment of the intensity of the circulation pattern inversion.
